# Bis{2-[(pyridin-2-yl)methyl­idene­amino]­benzoato-κ^3^ *N*,*N*′,*O*}chromium(III) nitrate monohydrate

**DOI:** 10.1107/S1600536814005649

**Published:** 2014-03-15

**Authors:** Elena A. Buvaylo, Vladimir N. Kokozay, Olga Yu. Vassilyeva, Brian W. Skelton

**Affiliations:** a Department of Inorganic Chemistry, Taras Shevchenko National University of Kyiv, 64/13 Volodymyrska Street, Kyiv 01601, Ukraine; b Centre for Microscopy, Characterisation and Analysis, University of Western Australia, 35 Stirling Highway, Crawley, WA 6009, Australia

## Abstract

The title complex salt hydrate, [Cr(C_13_H_9_N_2_O_2_)_2_]NO_3_·H_2_O, comprises discrete cations, nitrate anions and solvent water mol­ecules. The Cr^III^ atom is octa­hedrally coordinated by two anionic Schiff base ligands with the O atoms being *cis*. The two ligands differ significantly with dihedral angles between the pyridine and benzene rings of 4.8 (2) and 24.9 (2)°. The nitrate anion and solvent water mol­ecule were modelled as being disordered, with the major components having site-occupancy values of 0.856 (14) and 0.727 (16), respectively. The crystal is built of alternating layers of cations and of anions plus water mol­ecules, stacked along the *c* axis.

## Related literature   

For the synthesis of the Schiff base ligand and the structures of its complexes, see: Dey *et al.* (2003 ▶), Mukhopadhyay & Pal (2005 ▶); Sen *et al.* (2006 ▶).
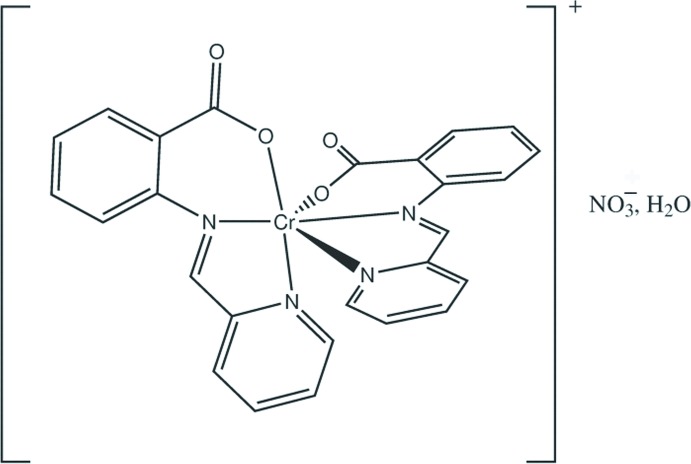


## Experimental   

### 

#### Crystal data  

[Cr(C_13_H_9_N_2_O_2_)_2_]NO_3_·H_2_O
*M* *_r_* = 582.47
Triclinic, 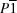

*a* = 7.9131 (11) Å
*b* = 11.4929 (15) Å
*c* = 13.5627 (18) Å
α = 86.105 (11)°
β = 79.290 (11)°
γ = 85.566 (11)°
*V* = 1206.5 (3) Å^3^
*Z* = 2
Cu *K*α radiation
μ = 4.47 mm^−1^
*T* = 100 K
0.11 × 0.05 × 0.03 mm

#### Data collection  

Oxford Diffraction Gemini diffractometer
Absorption correction: analytical (Clark & Reid, 1995 ▶) *T*_min_ = 0.681, *T*_max_ = 0.892
9601 measured reflections
4251 independent reflections
2790 reflections with *I* > 2σ(*I*)
*R*_int_ = 0.083

#### Refinement  

*R*[*F* ^2^ > 2σ(*F* ^2^)] = 0.057
*wR*(*F* ^2^) = 0.136
*S* = 0.99
4251 reflections
379 parameters
H-atom parameters constrained
Δρ_max_ = 0.43 e Å^−3^
Δρ_min_ = −0.27 e Å^−3^

### 

Data collection: *CrysAlis PRO* (Agilent, 2011 ▶); cell refinement: *CrysAlis PRO*; data reduction: *CrysAlis PRO*; program(s) used to solve structure: *SIR92* (Altomare *et al.*, 1994 ▶); program(s) used to refine structure: *SHELXL97* (Sheldrick, 2008 ▶); molecular graphics: *ORTEPII* (Johnson, 1976 ▶); software used to prepare material for publication: *WinGX* (Farrugia, 2012 ▶).

## Supplementary Material

Crystal structure: contains datablock(s) I, global. DOI: 10.1107/S1600536814005649/tk5300sup1.cif

Structure factors: contains datablock(s) I. DOI: 10.1107/S1600536814005649/tk5300Isup2.hkl

CCDC reference: 991354

Additional supporting information:  crystallographic information; 3D view; checkCIF report

## Figures and Tables

**Table 1:** Selected bond lengths (Å)

Cr1—O41	1.907 (3)
Cr1—O21	1.915 (3)
Cr1—N30	2.041 (3)
Cr1—N10	2.047 (3)
Cr1—N11	2.053 (3)
Cr1—N31	2.065 (3)

**Fig. 1.**
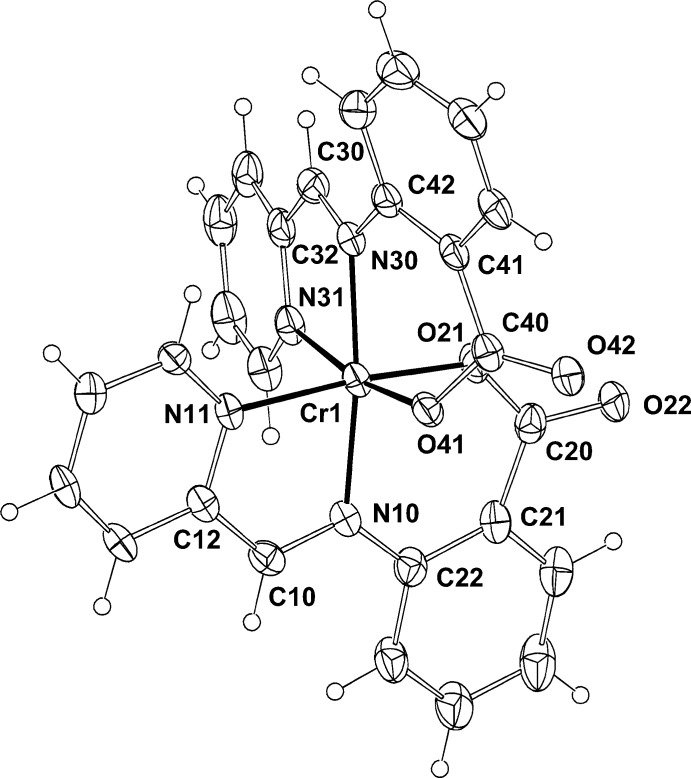
Molecular structure of the cation with the numbering scheme (the non-hydrogen atoms shown as 50% atomic displacement ellipsoids).

**Fig. 2.**
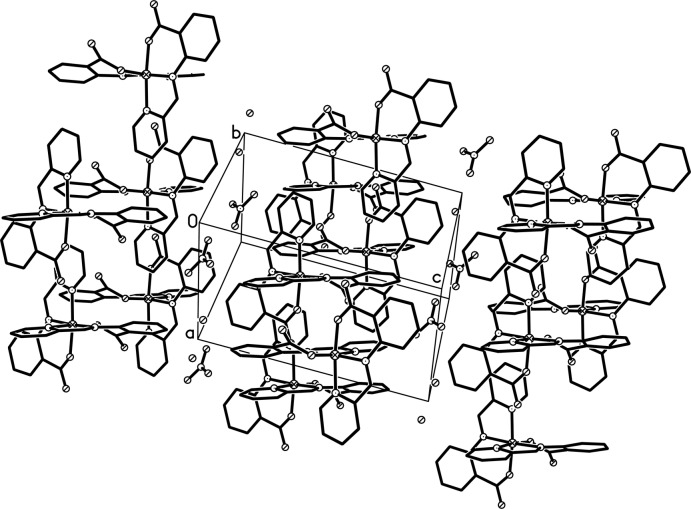
Packing diagram showing alternating layers of [CrL2]+ cations and nitrate anions plus solvent water molecules (C-bound H atoms and minor components of the disordered nitrate and water molecules are omitted for clarity).

## supplementary crystallographic information

### 2. Structural commentary

The title compound was prepared during studies of the coordination behaviour of the tridentate carboxyl­ate Schiff base ligand 2-*N*-(2'-pyridyl­imine)­benzoic acid (HL) which results from the condensation between 2-pyridine­carbaldehide and anthranilic acid. Known metal complexes containing deprotonated HL (Dey *et al.*, 2003; Mukhopadhyay *et al.*, 2005; Sen *et al.*, 2006) were prepared by *in situ* Schiff base synthesis - a synthetic approach utilized in the present work as well.

The title compound, Cr(C_13_H_10_N_2_O_2_)_2_NO_3_^.^H_2_O, is formed of discrete [Cr*L*_2_]^+^ cations, nitrate anions and solvent water molecules. The cation has no crystallographically imposed symmetry. The ligand molecules are deprotonated at the carboxyl­ato oxygen atom and coordinate to the Cr^III^ atom through the azomethine, pyridine-N and carboxyl­ato-O atoms in such a way that the metal atom is o­cta­hedrally surrounded by two anionic ligands with *cis* O atoms (Fig. 1 & Table 1). The Cr–N/O distances fall in the range 1.907 (3)–2.065 (3) Å, the *trans* angles at the metal atom lie in the range 170.57 (13)–173.61 (13), the *cis* angles vary from 80.47 (14) to 94.47 (14)°. The coordination geometry around the chromium centre is similar to that reported for Ni*L*_2_^.^H_2_O (Mukhopadhyay *et al.*, 2005).

The two ligands differ significantly. The atoms of ligand 1 are virtually coplanar with the dihedral angle between the pyridyl and benzene rings being 4.8 (2)°. By contrast, in ligand 2 this dihedral angle is 24.9 (2)°.

The crystal lattice is built of alternating layers of cations and of anions plus water molecules (Fig. 2).

### 5. Synthesis and crystallization

The ligand HL was prepared by refluxing 2-pyridine­carbaldehyde (0.38 ml, 4 mmol) with anthranilic acid (0.55 g, 4 mmol) in methanol (20 ml) for 0.5 h. The resultant yellow solution was left in open air overnight and used without further purification.

To a stirred methanol solution (10 ml) of Cr(NO_3_)_3_^.^9H_2_O (0.80 g, 2 mmol) in a 50 ml conic flask, HL in methanol from the previous preparation was added. The solution was magnetically stirred at 323 K for 20 minutes. The brown precipitate was filtered off. The red-brown solution was left to evaporate at room temperature. Red-brown rod-like microcrystals of the title compound were formed in a few days. They were collected by filter-suction, washed with dry Pr^i^OH and finally dried *in vacuo* (yield: 45%).

### 6. Refinement

The nitrate anion and solvent water molecule were modelled as being disordered. The site occupancy factors of the major component of the nitrate refined to 0.856 (14), and that of the disordered water molecule to 0.727 (16). Minor components of the disordered atoms were refined with isotropic displacement parameters. Water molecule hydrogen atoms were not located. All remaining hydrogen atoms were added at calculated positions (C—H = 0.95 Å) and refined by use of a riding model, with *U*_iso_(H) = 1.2*U*_eq_(parent atom).

### Crystal data

**Table:**

[Cr(C_13_H_9_N_2_O_2_)_2_]NO_3_·H_2_O	*Z* = 2
*M**_r_* = 582.47	*F*(000) = 598
Triclinic, *P*1	*D*_x_ = 1.603 Mg m^−^^3^
Hall symbol: -P 1	Cu *K*α radiation, λ = 1.54178 Å
*a* = 7.9131 (11) Å	Cell parameters from 1206 reflections
*b* = 11.4929 (15) Å	θ = 3.3–67.2°
*c* = 13.5627 (18) Å	µ = 4.47 mm^−^^1^
α = 86.105 (11)°	*T* = 100 K
β = 79.290 (11)°	Rod, red-brown
γ = 85.566 (11)°	0.11 × 0.05 × 0.03 mm
*V* = 1206.5 (3) Å^3^	

### Data collection

**Table:**

Oxford Diffraction Gemini diffractometer	4251 independent reflections
Graphite monochromator	2790 reflections with *I* > 2σ(*I*)
Detector resolution: 10.4738 pixels mm^-1^	*R*_int_ = 0.083
ω scans	θ_max_ = 67.0°, θ_min_ = 3.3°
Absorption correction: analytical (Clark & Reid, 1995)	*h* = −9→9
*T*_min_ = 0.681, *T*_max_ = 0.892	*k* = −11→13
9601 measured reflections	*l* = −10→16

### Refinement

**Table:**

Refinement on *F*^2^	Primary atom site location: structure-invariant direct methods
Least-squares matrix: full	Secondary atom site location: difference Fourier map
*R*[*F*^2^ > 2σ(*F*^2^)] = 0.057	Hydrogen site location: inferred from neighbouring sites
*wR*(*F*^2^) = 0.136	H-atom parameters constrained
*S* = 0.99	*w* = 1/[σ^2^(*F*_o_^2^) + (0.0496*P*)^2^] where *P* = (*F*_o_^2^ + 2*F*_c_^2^)/3
4251 reflections	(Δ/σ)_max_ = 0.002
379 parameters	Δρ_max_ = 0.43 e Å^−^^3^
0 restraints	Δρ_min_ = −0.27 e Å^−^^3^

### Special details

**Table:**

Geometry. All s.u.'s (except the s.u. in the dihedral angle between two l.s. planes) are estimated using the full covariance matrix. The cell s.u.'s are taken into account individually in the estimation of s.u.'s in distances, angles and torsion angles; correlations between s.u.'s in cell parameters are only used when they are defined by crystal symmetry. An approximate (isotropic) treatment of cell s.u.'s is used for estimating s.u.'s involving l.s. planes.
Refinement. Refinement of *F*^2^ against ALL reflections. The weighted *R*-factor *wR* and goodness of fit *S* are based on *F*^2^, conventional *R*-factors *R* are based on *F*, with *F* set to zero for negative *F*^2^. The threshold expression of *F*^2^ > 2σ(*F*^2^) is used only for calculating *R*-factors(gt) *etc*. and is not relevant to the choice of reflections for refinement. *R*-factors based on *F*^2^ are statistically about twice as large as those based on *F*, and *R*- factors based on ALL data will be even larger.The nitrate anion and solvent water molecule were modelled as being disordered. The site occupancy factors of the two components of the nitrate refined to 0.856 (14) and its complement. Those for the two components of the disordered water molecule appeared to be significantly different and were refined to 0.727 (16) and its complement. Minor components of the disordered atoms were refined with isotropic displacement parameters. Water molecule hydrogen atoms were not located.

### Fractional atomic coordinates and isotropic or equivalent isotropic displacement parameters (Å^2^)

**Table:**

	*x*	*y*	*z*	*U*_iso_*/*U*_eq_	Occ. (<1)
Cr1	0.54166 (9)	0.74547 (6)	0.61375 (5)	0.0373 (2)	
N11	0.7561 (4)	0.6436 (3)	0.6386 (2)	0.0358 (8)	
C12	0.7217 (6)	0.5754 (4)	0.7256 (3)	0.0413 (10)	
C13	0.8463 (6)	0.4967 (4)	0.7569 (3)	0.0477 (11)	
H13	0.821	0.4509	0.8182	0.057*	
C14	1.0056 (6)	0.4866 (4)	0.6976 (3)	0.0491 (11)	
H14	1.0916	0.4319	0.7167	0.059*	
C15	1.0425 (6)	0.5553 (4)	0.6101 (3)	0.0452 (10)	
H15	1.1539	0.5499	0.5692	0.054*	
C16	0.9120 (5)	0.6332 (4)	0.5828 (3)	0.0382 (9)	
H16	0.9362	0.6803	0.5222	0.046*	
C10	0.5485 (6)	0.5899 (4)	0.7818 (3)	0.0437 (10)	
H10	0.5167	0.5473	0.8442	0.052*	
N10	0.4359 (4)	0.6618 (3)	0.7463 (2)	0.0385 (8)	
C21	0.1464 (6)	0.7587 (4)	0.7554 (3)	0.0445 (10)	
C22	0.2644 (6)	0.6824 (4)	0.7983 (3)	0.0442 (10)	
C23	0.2101 (6)	0.6264 (5)	0.8926 (3)	0.0585 (13)	
H23	0.2878	0.5734	0.9215	0.07*	
C24	0.0447 (7)	0.6481 (6)	0.9433 (3)	0.0738 (17)	
H24	0.0101	0.6101	1.0075	0.089*	
C25	−0.0730 (7)	0.7238 (6)	0.9031 (4)	0.0772 (19)	
H25	−0.1872	0.7381	0.9387	0.093*	
C26	−0.0193 (6)	0.7777 (5)	0.8101 (4)	0.0597 (13)	
H26	−0.0987	0.8301	0.782	0.072*	
C20	0.1775 (6)	0.8256 (4)	0.6544 (3)	0.0457 (10)	
O21	0.3303 (4)	0.8357 (3)	0.6057 (2)	0.0465 (7)	
O22	0.0532 (4)	0.8712 (3)	0.6203 (3)	0.0635 (9)	
N31	0.6119 (5)	0.8854 (3)	0.6813 (2)	0.0418 (8)	
C32	0.6966 (6)	0.9635 (4)	0.6123 (3)	0.0467 (11)	
C33	0.7415 (6)	1.0696 (4)	0.6378 (4)	0.0548 (12)	
H33	0.7988	1.1224	0.588	0.066*	
C34	0.7011 (7)	1.0973 (4)	0.7376 (4)	0.0619 (14)	
H34	0.732	1.1692	0.7576	0.074*	
C35	0.6163 (7)	1.0201 (5)	0.8070 (4)	0.0620 (14)	
H35	0.5875	1.0384	0.8756	0.074*	
C36	0.5720 (6)	0.9140 (4)	0.7769 (3)	0.0538 (12)	
H36	0.5123	0.8612	0.8257	0.065*	
C30	0.7297 (6)	0.9282 (4)	0.5101 (3)	0.0449 (10)	
H30	0.7933	0.9752	0.4581	0.054*	
N30	0.6723 (4)	0.8323 (3)	0.4901 (2)	0.0397 (8)	
C41	0.5867 (5)	0.7161 (4)	0.3666 (3)	0.0399 (10)	
C42	0.6963 (5)	0.7961 (4)	0.3896 (3)	0.0419 (10)	
C43	0.8237 (6)	0.8424 (4)	0.3149 (3)	0.0547 (12)	
H43	0.9	0.8948	0.3316	0.066*	
C44	0.8384 (7)	0.8117 (5)	0.2164 (3)	0.0623 (14)	
H44	0.9261	0.842	0.1658	0.075*	
C45	0.7261 (7)	0.7375 (5)	0.1919 (3)	0.0571 (13)	
H45	0.7342	0.7187	0.124	0.069*	
C46	0.6018 (6)	0.6903 (4)	0.2656 (3)	0.0485 (11)	
H46	0.525	0.6393	0.2476	0.058*	
C40	0.4502 (6)	0.6555 (4)	0.4412 (3)	0.0415 (10)	
O41	0.4786 (4)	0.6332 (2)	0.53221 (18)	0.0402 (7)	
O42	0.3241 (4)	0.6254 (3)	0.4119 (2)	0.0503 (8)	
N1	0.4349 (7)	0.7154 (5)	1.0410 (3)	0.0656 (12)	
O11	0.5173 (8)	0.6206 (5)	1.0362 (4)	0.078 (2)	0.856 (14)
O12	0.2875 (9)	0.7275 (7)	1.0988 (3)	0.078 (2)	0.856 (14)
O13	0.4921 (9)	0.8027 (4)	0.9909 (3)	0.0713 (17)	0.856 (14)
O14	0.354 (3)	0.668 (2)	1.1045 (15)	0.031 (7)*	0.144 (14)
O15	0.561 (5)	0.656 (4)	0.982 (3)	0.089 (12)*	0.144 (14)
O16	0.389 (7)	0.816 (4)	0.995 (3)	0.092 (12)*	0.144 (14)
O1	0.1743 (16)	0.9903 (10)	0.9800 (8)	0.185 (6)	0.727 (16)
O2	0.129 (2)	0.9116 (18)	1.0468 (15)	0.100 (8)*	0.273 (16)

### Atomic displacement parameters (Å^2^)

**Table:**

	*U*^11^	*U*^22^	*U*^33^	*U*^12^	*U*^13^	*U*^23^
Cr1	0.0381 (4)	0.0373 (4)	0.0385 (3)	0.0040 (3)	−0.0158 (3)	0.0012 (3)
N11	0.037 (2)	0.0369 (19)	0.0365 (16)	0.0042 (15)	−0.0163 (15)	−0.0046 (14)
C12	0.046 (3)	0.040 (2)	0.040 (2)	0.003 (2)	−0.0167 (19)	−0.0007 (18)
C13	0.055 (3)	0.047 (3)	0.043 (2)	0.010 (2)	−0.021 (2)	0.0056 (19)
C14	0.048 (3)	0.049 (3)	0.052 (2)	0.016 (2)	−0.025 (2)	0.000 (2)
C15	0.040 (2)	0.048 (3)	0.050 (2)	0.005 (2)	−0.0144 (19)	−0.011 (2)
C16	0.044 (3)	0.036 (2)	0.0366 (19)	−0.0026 (18)	−0.0126 (18)	0.0002 (17)
C10	0.054 (3)	0.043 (2)	0.0354 (19)	−0.002 (2)	−0.0125 (19)	0.0000 (18)
N10	0.040 (2)	0.040 (2)	0.0370 (16)	0.0031 (16)	−0.0134 (15)	−0.0011 (15)
C21	0.041 (2)	0.049 (3)	0.047 (2)	0.005 (2)	−0.0178 (19)	−0.013 (2)
C22	0.045 (3)	0.056 (3)	0.034 (2)	−0.005 (2)	−0.0132 (18)	−0.0095 (19)
C23	0.047 (3)	0.091 (4)	0.039 (2)	0.004 (3)	−0.015 (2)	−0.004 (2)
C24	0.052 (3)	0.133 (6)	0.036 (2)	−0.003 (3)	−0.008 (2)	0.003 (3)
C25	0.045 (3)	0.136 (6)	0.050 (3)	0.012 (3)	−0.011 (2)	−0.024 (3)
C26	0.045 (3)	0.078 (4)	0.061 (3)	0.003 (3)	−0.020 (2)	−0.019 (3)
C20	0.041 (3)	0.034 (2)	0.067 (3)	−0.0011 (19)	−0.022 (2)	−0.006 (2)
O21	0.0393 (18)	0.0467 (18)	0.0559 (16)	0.0077 (14)	−0.0209 (14)	0.0026 (14)
O22	0.0394 (18)	0.057 (2)	0.094 (2)	0.0046 (16)	−0.0276 (17)	0.0244 (18)
N31	0.043 (2)	0.0366 (19)	0.0499 (19)	0.0056 (16)	−0.0218 (16)	−0.0053 (16)
C32	0.040 (2)	0.036 (2)	0.069 (3)	0.006 (2)	−0.028 (2)	0.003 (2)
C33	0.045 (3)	0.036 (3)	0.090 (3)	0.007 (2)	−0.033 (2)	−0.006 (2)
C34	0.059 (3)	0.041 (3)	0.096 (4)	0.013 (2)	−0.042 (3)	−0.018 (3)
C35	0.066 (3)	0.055 (3)	0.075 (3)	0.016 (3)	−0.037 (3)	−0.022 (3)
C36	0.053 (3)	0.055 (3)	0.058 (3)	0.008 (2)	−0.023 (2)	−0.007 (2)
C30	0.040 (2)	0.036 (2)	0.060 (3)	0.0017 (19)	−0.017 (2)	0.006 (2)
N30	0.0346 (19)	0.041 (2)	0.0443 (18)	0.0032 (16)	−0.0158 (15)	0.0069 (15)
C41	0.036 (2)	0.045 (2)	0.038 (2)	0.0094 (19)	−0.0125 (17)	0.0020 (18)
C42	0.035 (2)	0.044 (2)	0.045 (2)	0.0065 (19)	−0.0117 (18)	0.0069 (18)
C43	0.043 (3)	0.058 (3)	0.060 (3)	0.002 (2)	−0.008 (2)	0.007 (2)
C44	0.053 (3)	0.074 (4)	0.051 (3)	0.010 (3)	0.003 (2)	0.012 (2)
C45	0.054 (3)	0.070 (3)	0.043 (2)	0.016 (3)	−0.008 (2)	0.005 (2)
C46	0.049 (3)	0.049 (3)	0.046 (2)	0.019 (2)	−0.016 (2)	−0.001 (2)
C40	0.043 (3)	0.038 (2)	0.045 (2)	0.0071 (19)	−0.0155 (19)	−0.0049 (18)
O41	0.0452 (17)	0.0414 (16)	0.0371 (13)	−0.0019 (13)	−0.0171 (12)	0.0030 (11)
O42	0.0506 (19)	0.060 (2)	0.0462 (15)	−0.0083 (15)	−0.0226 (14)	0.0020 (14)
N1	0.085 (4)	0.070 (3)	0.047 (2)	−0.014 (3)	−0.024 (2)	0.006 (2)
O11	0.115 (4)	0.058 (3)	0.068 (4)	0.007 (3)	−0.040 (3)	0.002 (3)
O12	0.081 (4)	0.099 (5)	0.051 (2)	−0.014 (4)	0.001 (2)	−0.014 (3)
O13	0.067 (4)	0.077 (3)	0.063 (3)	0.005 (3)	−0.008 (2)	0.024 (2)
O1	0.227 (12)	0.165 (10)	0.144 (8)	0.048 (9)	−0.028 (8)	0.044 (7)

### Geometric parameters (Å, º)

**Table:**

Cr1—O41	1.907 (3)	N31—C36	1.334 (6)
Cr1—O21	1.915 (3)	N31—C32	1.369 (6)
Cr1—N30	2.041 (3)	C32—C33	1.380 (6)
Cr1—N10	2.047 (3)	C32—C30	1.442 (6)
Cr1—N11	2.053 (3)	C33—C34	1.385 (7)
Cr1—N31	2.065 (3)	C33—H33	0.95
N11—C16	1.323 (5)	C34—C35	1.367 (8)
N11—C12	1.367 (5)	C34—H34	0.95
C12—C13	1.391 (6)	C35—C36	1.401 (7)
C12—C10	1.443 (6)	C35—H35	0.95
C13—C14	1.365 (7)	C36—H36	0.95
C13—H13	0.95	C30—N30	1.287 (5)
C14—C15	1.377 (6)	C30—H30	0.95
C14—H14	0.95	N30—C42	1.427 (5)
C15—C16	1.400 (6)	C41—C42	1.400 (6)
C15—H15	0.95	C41—C46	1.403 (6)
C16—H16	0.95	C41—C40	1.513 (6)
C10—N10	1.304 (5)	C42—C43	1.398 (6)
C10—H10	0.95	C43—C44	1.387 (7)
N10—C22	1.418 (6)	C43—H43	0.95
C21—C26	1.390 (7)	C44—C45	1.375 (8)
C21—C22	1.409 (6)	C44—H44	0.95
C21—C20	1.513 (7)	C45—C46	1.380 (7)
C22—C23	1.401 (6)	C45—H45	0.95
C23—C24	1.373 (7)	C46—H46	0.95
C23—H23	0.95	C40—O42	1.222 (5)
C24—C25	1.386 (8)	C40—O41	1.300 (5)
C24—H24	0.95	N1—O14	1.11 (2)
C25—C26	1.375 (8)	N1—O11	1.225 (7)
C25—H25	0.95	N1—O13	1.239 (7)
C26—H26	0.95	N1—O12	1.282 (8)
C20—O22	1.229 (5)	N1—O15	1.33 (4)
C20—O21	1.275 (5)	N1—O16	1.33 (4)
			
O41—Cr1—O21	89.52 (12)	O22—C20—O21	120.4 (4)
O41—Cr1—N30	91.12 (13)	O22—C20—C21	119.0 (4)
O21—Cr1—N30	92.67 (13)	O21—C20—C21	120.6 (4)
O41—Cr1—N10	94.27 (13)	C20—O21—Cr1	131.4 (3)
O21—Cr1—N10	92.10 (14)	C36—N31—C32	118.1 (4)
N30—Cr1—N10	172.82 (14)	C36—N31—Cr1	129.7 (3)
O41—Cr1—N11	92.27 (12)	C32—N31—Cr1	111.8 (3)
O21—Cr1—N11	173.61 (13)	N31—C32—C33	122.8 (4)
N30—Cr1—N11	93.43 (14)	N31—C32—C30	114.8 (4)
N10—Cr1—N11	81.65 (14)	C33—C32—C30	122.4 (5)
O41—Cr1—N31	170.57 (13)	C32—C33—C34	118.4 (5)
O21—Cr1—N31	86.65 (13)	C32—C33—H33	120.8
N30—Cr1—N31	80.47 (14)	C34—C33—H33	120.8
N10—Cr1—N31	94.47 (14)	C35—C34—C33	119.3 (5)
N11—Cr1—N31	92.48 (13)	C35—C34—H34	120.3
C16—N11—C12	118.7 (3)	C33—C34—H34	120.3
C16—N11—Cr1	130.0 (3)	C34—C35—C36	120.0 (5)
C12—N11—Cr1	111.3 (3)	C34—C35—H35	120
N11—C12—C13	121.7 (4)	C36—C35—H35	120
N11—C12—C10	115.7 (4)	N31—C36—C35	121.4 (5)
C13—C12—C10	122.6 (4)	N31—C36—H36	119.3
C14—C13—C12	118.5 (4)	C35—C36—H36	119.3
C14—C13—H13	120.7	N30—C30—C32	119.4 (4)
C12—C13—H13	120.7	N30—C30—H30	120.3
C13—C14—C15	120.4 (4)	C32—C30—H30	120.3
C13—C14—H14	119.8	C30—N30—C42	121.3 (4)
C15—C14—H14	119.8	C30—N30—Cr1	113.3 (3)
C14—C15—C16	118.4 (4)	C42—N30—Cr1	125.3 (3)
C14—C15—H15	120.8	C42—C41—C46	117.7 (4)
C16—C15—H15	120.8	C42—C41—C40	125.8 (3)
N11—C16—C15	122.3 (4)	C46—C41—C40	116.5 (4)
N11—C16—H16	118.9	C43—C42—C41	120.7 (4)
C15—C16—H16	118.9	C43—C42—N30	120.6 (4)
N10—C10—C12	119.5 (4)	C41—C42—N30	118.7 (4)
N10—C10—H10	120.2	C44—C43—C42	119.8 (5)
C12—C10—H10	120.2	C44—C43—H43	120.1
C10—N10—C22	122.7 (4)	C42—C43—H43	120.1
C10—N10—Cr1	111.8 (3)	C45—C44—C43	120.1 (5)
C22—N10—Cr1	125.4 (3)	C45—C44—H44	119.9
C26—C21—C22	118.3 (4)	C43—C44—H44	119.9
C26—C21—C20	114.1 (4)	C44—C45—C46	120.3 (4)
C22—C21—C20	127.6 (4)	C44—C45—H45	119.9
C23—C22—C21	119.2 (4)	C46—C45—H45	119.9
C23—C22—N10	120.3 (4)	C45—C46—C41	121.3 (5)
C21—C22—N10	120.5 (4)	C45—C46—H46	119.4
C24—C23—C22	120.1 (5)	C41—C46—H46	119.4
C24—C23—H23	120	O42—C40—O41	123.6 (4)
C22—C23—H23	120	O42—C40—C41	118.6 (4)
C23—C24—C25	121.7 (5)	O41—C40—C41	117.6 (4)
C23—C24—H24	119.2	C40—O41—Cr1	124.6 (3)
C25—C24—H24	119.2	O11—N1—O13	121.1 (6)
C26—C25—C24	117.9 (5)	O11—N1—O12	120.9 (6)
C26—C25—H25	121	O13—N1—O12	118.0 (6)
C24—C25—H25	121	O14—N1—O15	119 (2)
C25—C26—C21	122.7 (5)	O14—N1—O16	126 (2)
C25—C26—H26	118.6	O15—N1—O16	111 (2)
C21—C26—H26	118.6		
